# Outcomes of MagLev LVAD Support in Patients Requiring Preoperative Continuous Renal Replacement Therapy

**DOI:** 10.3390/jcm14238502

**Published:** 2025-11-30

**Authors:** Christopher L. He, Clayton J. Rust, Ian M. Kusher, Sally El Sammak, Ailin Tang, Joshua D. Preston, Supreet S. Randhawa, Michael E. Halkos, Muath M. Bishawi, Mani A. Daneshmand, Joshua L. Chan

**Affiliations:** Division of Cardiothoracic Surgery, Department of Surgery, Emory University School of Medicine, Atlanta, GA 30345, USA

**Keywords:** magnetically levitated left ventricular assist device (MagLev LVAD), continuous renal replacement therapy (CRRT), acute kidney injury (AKI), advanced heart failure, survival outcomes, perioperative mortality

## Abstract

**Background/Objectives**: Acute Kidney Injury (AKI) requiring continuous renal replacement therapy (CRRT) has historically been a contraindication for left ventricular assist device (LVAD) implantation. However, advancements in magnetically levitated (MagLev) LVADs warrant reevaluation. **Methods**: A retrospective review of adult LVAD recipients at a tertiary center (2009–2024) was performed. Patients were stratified by preoperative CRRT status and LVAD type. Baseline characteristics and perioperative morbidity, Kaplan–Meier survival estimates, restrictive mean survival time (RMST), and Cox proportional hazards models were assessed. **Results**: Among 312 MagLev LVAD recipients, 22 (7.1%) required preoperative CRRT. Compared to non-CRRT patients, the CRRT group had higher illness severity (INTERMACS 1 or 2: 95% vs. 71%, *p* = 0.019). Despite this, preoperative CRRT was not associated with worse mortality within the MagLev cohort at 30 days (9.1% vs. 7.9%), 1 year (18.2% vs. 17.9%), or 2 years (31.8% vs. 20.7%; *p* = 0.31). RMST at 1 year was also similar (305 vs. 311 days; *p* = 0.85). In contrast, patients on CRRT receiving non-MagLev devices had significantly worse outcomes, with 30-day, 1-year, and 2-year mortality rates of 57.1%, 71.4%, and 78.6%, respectively. RMST analysis showed a 170-day survival advantage at 1 year for MagLev vs. non-MagLev CRRT patients (*p* < 0.01). **Conclusions**: In this single-center cohort, preoperative CRRT was not associated with higher mortality among MagLev LVAD recipients and may challenge traditional contraindications against LVAD therapy. Further investigations using larger cohorts are necessary to further evaluate these findings and delineate patient subgroups that may derive the greatest clinical benefit.

## 1. Introduction

Left ventricular assist devices (LVADs) are a life-saving treatment option for patients with advanced heart failure [1,2,3,4]. Acute kidney injury (AKI), particularly in patients requiring continuous renal replacement therapy (CRRT), is a known risk factor for increased mortality following LVAD implantation [5,6,7,8]. The mechanisms contributing to this elevated risk are multifactorial and include perioperative hemodynamic instability, right ventricular failure, renal venous congestion, and ischemic or inflammatory injury leading to impaired renal recovery [3,4,5,9,10]. International guidelines align with this data and classify dialysis as a Class III contraindication for LVAD use as destination therapy [11].

In recent years, advancements in CRRT technology and the introduction of magnetically levitated (MagLev) LVADs have reshaped the management of advanced heart failure [1]. MagLev devices use a fully suspended rotor without mechanical bearings, reducing friction and shear stress on circulating blood [12,13]. Compared with older axial-flow pumps, this design improves hemocompatibility and durability, lowering the risk of pump thrombosis and hemolysis [1,13,14]. Although all second- and third-generation LVADs provide continuous rather than truly pulsatile flow, MagLev systems incorporate a rapid, programmed rotor speed modulation that creates an artificial pulse to reduce pump stasis [1,14]. These design features enhance hemodynamic stability and may help mitigate end-organ injury, including acute kidney injury (AKI), in appropriately selected patients [3,4,9]. Careful patient selection and optimization strategies have demonstrated improved 1-year survival in patients with severe renal insufficiency undergoing LVAD implantation [11,15]. However, data on outcomes in MagLev LVAD recipients dependent on CRRT preoperatively remains limited. The current study provides a contemporary analysis of survival and clinical trajectories in this high-risk cohort, offering insights into the use of modern LVAD therapy for critically ill patients. This study was motivated by institutional observations and prior reports suggesting that some patients with preoperative CRRT dependence may achieve meaningful recovery following MagLev LVAD implantation compared to prior generation LVADs. We hypothesized that the enhanced hemocompatibility and stable flow profile of MagLev devices could improve perioperative tolerance and survival in this high-risk population.

## 2. Materials and Methods

This single-center, retrospective cohort study evaluated all adults (≥18 years) who underwent LVAD implantation at Emory University Hospital, a tertiary academic center, between February 2009 and February 2024 using the Society of Thoracic Surgeons (STS) Interagency Registry for Mechanically Assisted Circulatory Support (INTERMACS) and Adult Cardiac Surgery (ACSD) databases. All patients meeting these criteria were included, with no exclusion criteria applied. Patients were stratified into groups by preoperative CRRT requirement and LVAD type (MagLev vs. non-MagLev). Baseline demographics and additional clinical and operative characteristics were obtained from these databases. The primary outcome measure was mortality at 30 days, 1 year, and 2 years post-implantation. Secondary outcomes included perioperative morbidity.

Preoperative characteristics included age, body mass index (BMI), comorbidities, New York Heart Association (NYHA) Class, INTERMACS profile, cardiac output, left ventricular end-diastolic diameter (LVEDD), right ventricular function, device strategy, and baseline renal function. Baseline renal function was assessed using the most recent serum creatinine value obtained either prior to or during the index hospitalization, and before device implantation, CRRT initiation, and the onset of AKI. Estimated glomerular filtration rate (eGFR) was calculated using the CKD-EPI 2021 equation and summarized as median and interquartile range. The INTERMACS profile was used as a validated marker for patient prognosis [16]. Perioperative morbidity was assessed during the index hospitalization and included stroke (neurological deficit persisting for more than 24 h), reoperation for persistent bleeding, and stage 3 AKI. Postoperative acute kidney injury (AKI) was defined based on STS database criteria indicating new initiation of renal replacement therapy during the index hospitalization, which correspond to the Kidney Disease: Improving Global Outcomes (KDIGO) stage 3 AKI classification [17]. Patients known to be receiving preoperative dialysis were identified; however detailed data were not available to determine whether these patients had end-stage kidney disease on chronic dialysis or were dually listed for combined heart-kidney transplantation. Additional postoperative complications included mechanical ventilation greater than 24 h.

Descriptive statistics were used to summarize demographic data. Continuous variables were reported as median ± interquartile range, while categorical variables were summarized as frequencies and percentages. Group comparisons were conducted using Wilcoxon rank sum, chi-square, or Fisher exact tests as appropriate. Kaplan–Meier survival analysis with log-rank testing was used to compare survival at 30 days, 1 year, and 2 years; cumulative mortality (events/N) was also reported, and restricted mean survival time (RMST) was calculated to account for the small sample size. Multivariable Cox proportional hazards models were constructed to assess the association between preoperative CRRT use and overall mortality up to two years among MagLev recipients. Covariates included INTERMACS 1–2 profile (disease severity), ECMO use, renal function (serum creatinine and BUN), and hemodynamic parameters (LVEDD and cardiac output). Inclusion of further covariates was limited due to model instability given the limited sample size. All statistical analyses and figure generations were performed using RStudio (R version 4.3.1; R Foundation for Statistical Computing, Vienna, Austria). This was a retrospective evaluation of de-identified data and the Institutional Review Board (IRB) granted a waiver of informed consent based on the provided documentation.

## 3. Results

### 3.1. Preoperative Characteristics of MagLev Patients with vs. Without CRRT

As shown in Table 1, a total of 312 patients underwent MagLev LVAD implantation, including 22 (7.1%) who required preoperative CRRT. An additional 220 patients received non-MagLev LVADs over the specified time period, of which, 14 (6.4%) required preoperative CRRT. Compared to non-CRRT MagLev patients (290 patients), the MagLev CRRT group had higher acuity, reflected by a higher prevalence of INTERMACS Profiles 1 and 2 (95% vs. 71%, *p* = 0.019). Hemodynamically, they had higher native cardiac output (5.02 vs. 4.02 L/min, *p* < 0.011) prior to MagLev implantation. They were also more likely to have received preoperative mechanical circulatory support with ECMO (24% vs. 5.5%, *p* = 0.009). No significant difference was seen in right ventricular function between the two groups, with the majority having mild to moderate dysfunction. The baseline characteristics and preoperative clinical variables are summarized in Table 1.

### 3.2. Peri-Operative Patient Morbidity

No significant differences were seen in rates of stroke, AKI, reoperation for bleeding, mechanical ventilation greater than 24 h, or deep sternal wound infections between MagLev LVAD compared to non-MagLev LVAD recipients (Table 2). Within the MagLev cohort, perioperative complication rates also did not differ significantly between CRRT-dependent and non-CRRT patients. New postoperative dialysis was required in 8 (2.7%) of MagLev LVAD patients without preoperative CRRT. Additional baseline characteristics and postoperative outcomes for the non-MagLev, non-CRRT cohort, which were not part of the primary analysis, are provided in the Supplementary Materials (Tables S1 and S2).

### 3.3. Long-Term Mortality and Post-Operative Renal Function

**Figure 1:**
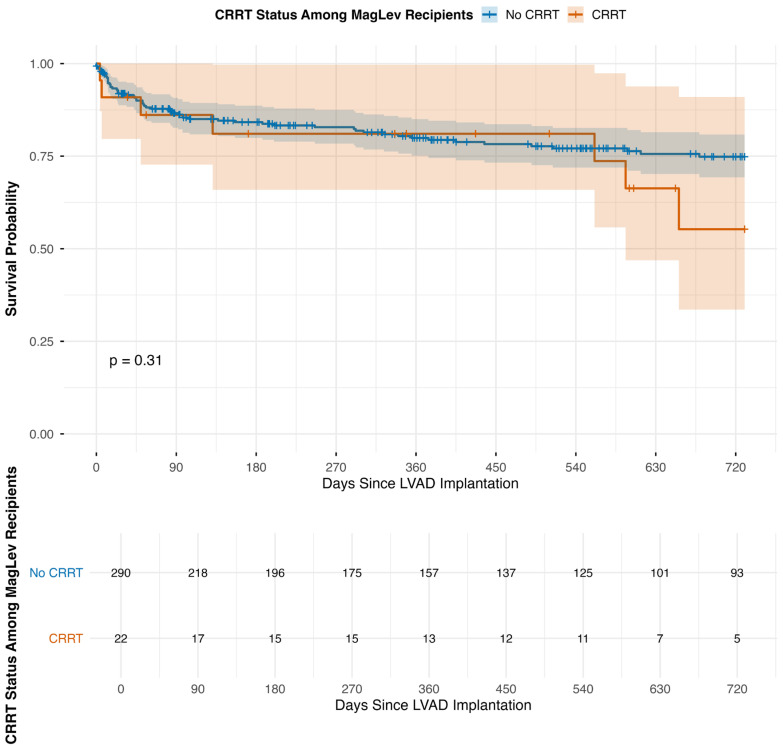
Survival of MagLev LVAD patients stratified by pre-implantation CRRT dependence at 30 days, 1 year, and 2 years.

Kaplan–Meier survival curves (Figure 1) demonstrated no significant differences in all-cause mortality between MagLev LVAD patients with and without preoperative CRRT. At 30 days, mortality was 9.1% in the CRRT group compared with 7.9% in the non-CRRT group, 18.2% versus 17.9% at 1 year, and 31.8% versus 20.7% at 2 years (*p* = 0.31). While these comparisons did not reach statistical significance, it is important to interpret them in the context of the small CRRT + MagLev cohort (n = 22), which led to wide confidence intervals and limited statistical power to detect modest differences in long-term outcomes. Cumulative incidence estimates are shown in Table 3.

**Table 3:** Cumulative Incidence Estimates of 30-Day, 1-Year, and 2-Year Mortality Stratified by LVAD Type and CRRT Status.

Group	Total Number of Patients	Deaths at 30 Days	Deaths at 1 Year	Deaths at 2 Years	30-Day Mortality (Incident/N) (%)	1-Year Mortality (Incident/N) (%)	2-Year Mortality (Incident/N) (%)
**A. MagLev LVAD Patients by CRRT Status**							
MagLev (Non-CRRT)	290	23	52	60	7.9%	17.9%	20.7%
MagLev + CRRT	22	2	4	7	9.1%	18.2%	31.8%
**B. All CRRT Recipients by LVAD Type**							
Non-MagLev + CRRT	14	8	10	11	57.1%	71.4%	78.6%
MagLev + CRRT	22	2	4	7	8.7%	17.4%	30.4%

**Figure 2:**
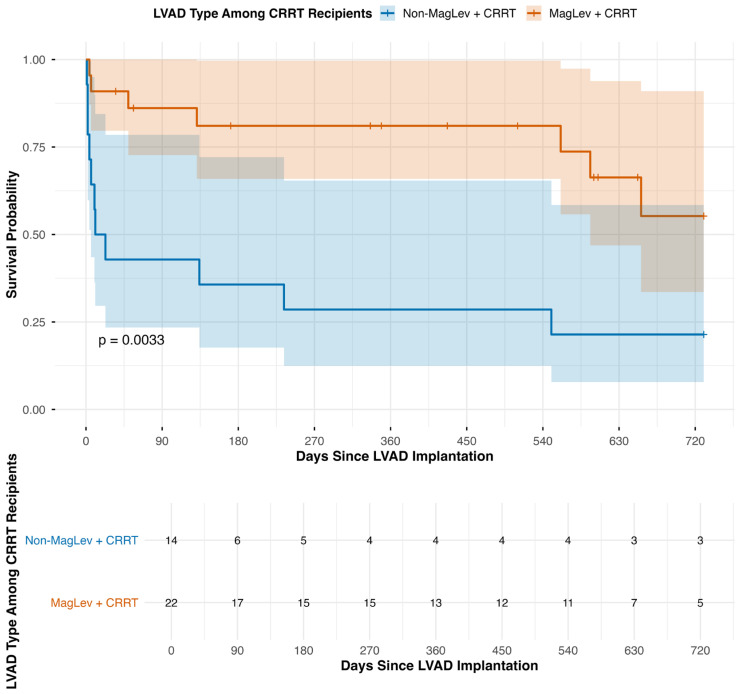
Survival of LVAD patients (both MagLev and non-MagLev) stratified by pre-implantation CRRT dependence at 30 days, 1 year, and 2 years after device implantation.

The restricted RMST for CRRT versus non-CRRT MagLev patients was similar across time points: 27.7 vs. 28.5 days at 30 days (Δ = −0.8; *p* = 0.61), 305 vs. 311 days at 1 year (Δ = −5; *p* = 0.85), and 571 vs. 592 days at 2 years (Δ = −21; *p* = 0.72). Meanwhile, among patients requiring CRRT, non-MagLev devices were associated with substantially higher mortality than MagLev devices (30-day: 57.1% vs. 8.7%; 1-year: 71.4% vs. 17.4%; 2-year: 78.6% vs. 30.4%; Figure 2). Correspondingly, RMST values were lower for non-MagLev devices—17.1 vs. 27.7 days at 30 days, 135 vs. 305 days at 1 year, and 226 vs. 571 days at 2 years (Δ = 11, 171, and 345 days; all *p* < 0.01).

In the multivariable Cox proportional hazards model restricted to MagLev recipients (Table 4), the need for CRRT was not associated with increased mortality (HR = 1.22, 95%: CI 0.50–2.95; *p* = 0.66). Higher pre-operative creatinine was independently associated with worse survival (HR = 0.59, 95% CI: 0.35–0.99; *p* = 0.045), while other covariates, including INTERMACS 1–2 profile, ECMO use, BUN, LVEDD, and cardiac output, were not significant predictors. Additional data, including annual LVAD implantation by device type and mortality outcomes stratified by device strategy, are provided in the Supplementary Materials (Tables S3 and S4).

## 4. Discussion

The current study suggests that patients requiring continuous renal replacement therapy (CRRT) may be potential candidates for LVAD therapy, particularly with magnetically levitated devices. Historically, severe renal dysfunction has been associated with poor outcomes following LVAD implantation, leading guidelines to discourage its use as destination therapy for these patients [2,8,10,18]. However, our data indicate that MagLev LVAD recipients dependent on CRRT at time of implantation can achieve morbidity and survival rates comparable to those of non-CRRT recipients and is markedly a clear departure from outcomes observed in patients with older-generation (non-MagLev) LVADs.

The survival benefit observed in CRRT patients with MagLev LVADs compared to non-MagLev LVADs may be attributable to several factors. In the five-year follow-up of the landmark MOMENTUM 3 trial, MagLev LVAD recipients demonstrated lower rates of hemocompatibility-related adverse events (e.g., nonsurgical bleeding, thromboembolic events, pump thrombosis, neurologic complications) and lower overall mortality (hazard ratio: 0.72) relative to those with earlier generation axial-flow pumps (non-MagLev) [1,12,13]. The improved outcomes with MagLev LVADs are attributed primarily to their fully levitated rotor design, which eliminates mechanical bearings and minimizes blood–surface interaction, thereby reducing shear stress and thrombogenic potential [12,13,14]. These design improvements optimize flow paths, increase blood-flow gaps, and enhance surface hemodynamics, contributing to an improved hemocompatibility profile and better clinical outcomes compared with earlier-generation devices [19,20].

Renal recovery after LVAD implantation has been described for patients with acute or chronic cardiorenal syndrome, wherein reduced cardiac output, venous congestion, and neurohormonal imbalance exacerbate kidney dysfunction [3,4,9,10]. By augmenting cardiac output and reducing central venous pressures, LVADs often improve renal perfusion in those whose renal impairment is primarily driven by compromised hemodynamics rather than intrinsic kidney pathology.

Despite the encouraging results, several limitations warrant consideration. Firstly, this is a single-institution retrospective study, raising the possibility of selection bias, incomplete data acquisition, and restricted generalizability. Additionally, the historical practice of avoiding LVAD implantation in patients with significant renal impairment likely contributed to the relatively small proportion of CRRT-dependent individuals in our cohort, limiting the power for more granular subgroup analyses. The present study also used serum creatinine-based criteria to define AKI, as urine output data were unavailable. Consequently, milder or transient cases of AKI may have been underdiagnosed, potentially leading to an underestimation of the overall incidence of perioperative AKI among patients not receiving CRRT. Finally, our data sources may not have consistently differentiated acute-on-chronic kidney failure from isolated acute kidney injury, which could have influenced the observed patterns of renal recovery after implantation. Importantly, as an observational study, these findings demonstrate associations rather than causation, and should therefore be interpreted as hypothesis-generating. Although this study reports one of the largest contemporary experiences of MagLev LVAD use in CRRT-dependent patients, the overall sample size remains small, emphasizing the need for additional multicenter studies with larger cohorts, and ultimately, prospective clinical trials to better define outcomes and guide management strategies for patients with advanced kidney disease undergoing LVAD implantation. Moreover, these future investigations could better identify subpopulations within this high-risk group that may derive the most benefit, as well as those for whom LVAD therapy may be less advantageous. Finally, long-term outcomes beyond two years were not captured in sufficient detail, underscoring the importance of extended follow-up to determine the durability of clinical benefits and to characterize potential late complications in this vulnerable population.

## 5. Conclusions

This study suggests that CRRT dependence does not necessarily portend adverse outcomes in patients undergoing MagLev LVAD implantation. Improvements in device technology and a growing understanding of the interplay between cardiac and renal pathophysiology appear to have expanded the range of patients eligible for durable mechanical support. With growing experience and ongoing device advancements, LVAD therapy could become a more feasible option for patients with severe renal dysfunction, though the evidence remains limited. Further research should investigate which patients on renal replacement therapy may be appropriate candidates for LVAD implantation, incorporating comprehensive risk stratification analyses that consider kidney disease history, comorbidities, and organ dysfunction to better inform patient selection and optimize outcomes.

## Figures and Tables

**Table 1:** Baseline characteristics prior to implantation among magnetically levitated left ventricular (MagLev) device implantation recipients.

Baseline Characteristic	Non-CRRT N = 290 ^1^	CRRT N = 22 ^1^	*p*-Value ^2^
Age (years)	51.56 ± 12.78	48.35 ± 14.01	0.431
Sex (Male %)			0.630
Female	83 (29%)	5 (23%)	
Male	202 (71%)	17 (77%)	
Missing	5	0	
BMI (kg/m^2^)	28.65 ± 8.43	27.61 ± 5.70	0.936
Missing	0	1	
Diabetes Mellitus	127 (45%)	7 (32%)	0.273
Missing	5	0	
Hypertension	222 (78%)	15 (68%)	0.297
Missing	5	0	
Chronic Lung Disease			0.037
No	143 (49%)	10 (45%)	
Mild/Moderate	110 (38%)	6 (27%)	
Severe	22 (7.6%)	6 (27%)	
Other/Unknown	15 (5.2%)	0 (0%)	
Cerebrovascular Disease	81 (28%)	7 (32%)	0.807
Missing	5	0	
Peripheral Arterial Disease	12 (4.2%)	3 (14%)	0.083
Missing	5	0	
Immunocompromised State	16 (5.6%)	3 (14%)	0.146
Missing	5	0	
***Renal Characteristics***			
Creatinine (mg/dL) *	1.65 ± 0.96	1.95 ± 0.85	0.032
BUN (mmol/L)	11.76 ± 6.80	8.50 ± 6.51	0.005
Missing	0	1	
eGFR (mL/min/1.73 m^2^)	53 (38, 76)	40 (32, 66)	0.074
eGFR Category (mL/min/1.73 m^2^)			0.2
≥60	118 (41%)	7 (32%)	
45–59	63 (22%)	2 (9.1%)	
30–44	68 (24%)	9 (41%)	
<30	36 (13%)	4 (18%)	
Unknown	5	0	
***Cardiac Characteristics***			
Heart Failure Etiology			0.115
Congenital/Other	3 (1.0%)	0 (0%)	
Ischemic	60 (21%)	5 (23%)	
Non-ischemic	218 (75%)	14 (64%)	
Other Cardiomyopathy	4 (1.4%)	1 (4.5%)	
Unknown	5 (1.7%)	2 (9.1%)	
NYHA Class III or IV			0.067
No	19 (6.6%)	4 (18%)	
Yes	271 (93%)	18 (82%)	
INTERMACS Profile 1–2			0.019
No	83 (29%)	1 (4.8%)	
Yes	207 (71%)	20 (95%)	
Missing	0	1	
LVEDD (cm)	6.80 ± 1.13	6.33 ± 0.91	0.020
Missing	1	1	
RVEF Group			0.662
Normal	51 (18%)	2 (9.1%)	
Mild/Moderate	156 (54%)	12 (55%)	
Severe	38 (13%)	3 (14%)	
Unknown	45 (16%)	5 (23%)	
Cardiac Output (L/min)	4.02 ± 1.19	5.02 ± 1.74	0.011
Missing	32	2	
Cardiac Arrhythmia			0.372
No	94 (32%)	8 (36%)	
Yes	132 (46%)	12 (55%)	
Unknown	64 (22%)	2 (9.1%)	
Resuscitation ≤ 1 h			0.312
No	281 (99%)	21 (95%)	
Yes—Within 1 h of the start of the procedure	4 (1.4%)	1 (4.5%)	
Missing	5	0	
***Preoperative MCS***			
ECMO	16 (5.5%)	5 (24%)	0.009
Missing	0	1	
IABP	34 (12%)	4 (19%)	0.304
Missing	0	1	
RVAD	3 (1.0%)	1 (4.8%)	0.245
Missing	0	1	
LVAD Planned Strategy			0.083
Bridge to Transplant	88 (30%)	7 (32%)	
Destination Therapy	202 (70%)	14 (64%)	
Other/Unknown	0 (0%)	1 (4.5%)	

^1^ Data are presented as mean ± standard deviation, median (interquartile range), or number (percentage), as appropriate. ^2^ Wilcoxon rank sum test; Fisher’s exact test. * Preoperative creatinine was collected prior to device implantation, CRRT, and onset of AKI. Abbreviations: BMI, body mass index; BUN, blood urea nitrogen; CRRT, continuous renal replacement therapy; ECMO, extracorporeal Membrane Oxygenation; eGFR, estimated glomerular filtration rate; IABP, intra-aortic balloon pump; INTERMACS, Interagency Registry for Mechanically Assisted Circulatory Support; LVEDD, left ventricular end-diastolic diameter; NYHA, New York Heart Association; RVEF, right ventricular ejection fraction; RVAD, right ventricular assist device.

**Table 2:** Peri-operative morbidity among MagLev LVAD implantation recipients stratified by device generation and preoperative CRRT usage.

(**A**) MagLev LVADs: CRRT vs. Non-CRRT			
**Outcome**	**Non-CRRT** **N = 290 ^1^**	**CRRT** **N = 22 ^1^**	***p*-Value ^2^**
Stroke	13 (5.8%)	1 (5.3%)	>0.999
Reoperation due to bleeding	19 (8.5%)	1 (5.3%)	>0.999
Stage 3 AKI *	58 (26%)	—	—
Mechanical ventilation > 24 h	133 (60%)	14 (74%)	0.328
Postoperative Dialysis Requirement	44 (76%)	2 (67%)	>0.999
(**B**) CRRT Cohort: MagLev vs. Non-MagLev			
**Outcome**	**Non-MagLev** **N = 14 ^1^**	**MagLev** **N = 22 ^1^**	***p*-Value ^2^**
Stroke	1 (11%)	1 (5.3%)	>0.999
Reoperation due to bleeding	2 (22%)	1 (5.3%)	0.234
Stage 3 AKI *	0 (0%)	—	—
Mechanical ventilation > 24 h	6 (67%)	14 (74%)	>0.999
Postoperative Dialysis Requirement	0 (0%)	2 (67%)	>0.999

^1^ n = (%). ^2^ Fisher’s exact test. * Captures new postoperative dialysis initiation. This variable is not applicable to patients receiving preoperative CRRT, who by definition already met the criteria for KDIGO Stage 3 AKI prior to implantation.

**Table 4:** Multivariable Cox Proportional Hazards Model for Mortality Among MagLev Recipients.

Variable	Hazard Ratio (95% CI)	*p*-Value
Preoperative CRRT	1.22 (0.50–2.95)	0.6605
INTERMACS 1–2	1.71 (0.92–3.16)	0.0885
Preoperative ECMO	0.96 (0.34–2.70)	0.9427
Preoperative Creatinine	0.59 (0.35–0.99)	0.0448
BUN (mmol/L)	1.05 (0.99–1.11)	0.0790
LVEDD (cm)	0.87 (0.69–1.10)	0.2382
Cardiac Output (L/min)	1.17 (0.97–1.41)	0.1076

## Data Availability

The original contributions presented in this study are included in the article/Supplementary Materials. Further inquiries can be directed to the corresponding author.

## Supplementary Materials

The following supporting information can be downloaded at: https://www.mdpi.com/article/10.3390/jcm14238502/s1.

## Abbreviations

The following abbreviations are used in this manuscript:

ACSD	Adult Cardiac Surgery Database
AKI	Acute Kidney Injury
BMI	Body mass index
BUN	Blood urea nitrogen
CRRT	Continuous renal replacement therapy
ECMO	Extracorporeal Membrane Oxygenation
eGFR	Estimated glomerular filtration rate
IABP	Intra-aortic balloon pump
INTERMACS	Interagency Registry for Mechanically Assisted Circulatory Support
KDIGO	Kidney Disease: Improving Global Outcomes
LVAD	Left ventricular assist device
LVEDD	Left ventricular end-diastolic diameter
MagLev	Magnetically levitated
MCS	Mechanical circulatory support
NYHA	New York Heart Association
RVAD	Right ventricular assist device
RVEF	Right ventricular ejection fraction
STS	Society of Thoracic Surgeons
