# Supplementary material for: Outcomes of MagLev LVAD Support in Patients Requiring Preoperative Continuous Renal Replacement Therapy
Source: J Clin Med. 2025 Nov 30;14(23):8502. doi: 10.3390/jcm14238502 (PMC12693132; doi:10.3390/jcm14238502)
Supplement: Supplementary file 1 [file jcm-14-08502-s001.zip › Table S1.pdf]

Table S1. Baseline characteristics prior to implantation among non-MagLev, non-CRRT patients

**Baseline Characteristic**	**N = 207[1]**
**Age (years)**	50 ± 13
**Sex (Male %)**	
Female	59 (29%)
Male	148 (71%)
**BMI (kg/m²)**	28.1 ± 6.2
**Diabetes**	28 (32%)
**Hypertension**	63 (72%)
**NYHA Class**	
Class III–IV	176 (85%)
Unknown	24 (12%)
**Chronic Lung Disease**	
No	60 (29%)
Mild/Moderate	19 (9.2%)
Severe	8 (3.9%)
Other/Unknown	120 (58%)
**Cerebrovascular Disease**	20 (23%)
**Peripheral Arterial Disease**	5 (5.7%)
**Immunocompromised State**	3 (3.4%)
Renal Characteristics	
**Creatinine (mg/dL)***	1.72 ± 0.79
**BUN (mmol/L)**	34 ± 21
Cardiac Characteristics	
**RVEF Group**	
Normal	20 (14%)
Mild	15 (10%)
Moderate	49 (34%)
Severe	60 (42%)
**Cardiac Output (L/min)**	3.67 ± 1.17
**Cardiac Arrhythmia**	32 (78%)
**Resuscitation ≤ 1 hour**	
No	86 (99%)
Yes - Within 1 hour of the start of the procedure	1 (1.1%)
Preoperative MCS	
**ECMO**	7 (3.4%)
**IABP**	30 (34%)
**RVAD**	
No	207 (100%)
**LVAD Planned Strategy**	
Bridge to Transplant	138 (67%)
Destination Therapy	68 (33%)
Other / Unknown	1 (0.5%)

[1]Mean ± SD; n (%)
