# Supplementary material for: Outcomes of MagLev LVAD Support in Patients Requiring Preoperative Continuous Renal Replacement Therapy
Source: J Clin Med. 2025 Nov 30;14(23):8502. doi: 10.3390/jcm14238502 (PMC12693132; doi:10.3390/jcm14238502)
Supplement: Supplementary file 1 [file jcm-14-08502-s001.zip › Table S2.pdf]

Table S2. Peri-operative morbidity among following time of non-MagLev LVAD implantation in patients without preoperative CRRT requirement

Outcome	N = 207[1]
**Stroke**	**3 (4.7%)**
**Reoperation due to bleeding**	**6 (9.4%)**
**Stage 3 AKI***	**14 (22%)**
**Mechanical ventilation > 24 hours**	**45 (70%)**
**Postoperative Dialysis Requirement**	**10 (71%)**

[1]n (%)
