# Supplementary material for: Outcomes of MagLev LVAD Support in Patients Requiring Preoperative Continuous Renal Replacement Therapy
Source: J Clin Med. 2025 Nov 30;14(23):8502. doi: 10.3390/jcm14238502 (PMC12693132; doi:10.3390/jcm14238502)
Supplement: Supplementary file 1 [file jcm-14-08502-s001.zip › Table S3.pdf]

Table S3. Annual LVAD Implantations by Device Type

Implant Year	HeartWare HVAD	HeartMate 3 (MagLev)	Total Implants
2,008	1	0	1
2,009	2	0	2
2,010	1	0	1
2,011	1	0	1
2,013	1	0	1
2,015	2	0	2
2,016	2	0	2
2,017	3	0	3
2,018	1	5	6
2,019	0	34	34
2,020	0	47	47
2,021	0	70	70
2,022	0	83	83
2,023	0	65	65
2,024	0	8	8
