# Supplementary material for: Outcomes of MagLev LVAD Support in Patients Requiring Preoperative Continuous Renal Replacement Therapy
Source: J Clin Med. 2025 Nov 30;14(23):8502. doi: 10.3390/jcm14238502 (PMC12693132; doi:10.3390/jcm14238502)
Supplement: Supplementary file 1 [file jcm-14-08502-s001.zip › Table S4.pdf]

Table S4. Stratified Cox Proportional Hazards Models for Overall Mortality (2 years) by Device Strategy

Variable	Bridge-to-Transplant (n = 85, 4 deaths)	Destination Therapy (n = 191, 10 deaths)
CRRT (Yes vs No)	HR = 5.52 (0.17–178.6), p = 0.33	HR = 34.47 (4.74–250.8), p < 0.001
INTERMACS 1–2	HR = 8.47e8 (0–∞), p = 0.99	HR = 0.64 (0.10–4.06), p = 0.64
ECMO	HR = 1.86e–8 (0–∞), p = 0.99	HR = 2.64 (0.48–14.5), p = 0.27
Creatinine	HR = 0.07 (0.001–3.54), p = 0.18	HR = 0.87 (0.19–4.03), p = 0.86
BUN	HR = 1.15 (0.89–1.49), p = 0.27	HR = 1.03 (0.88–1.19), p = 0.73
LVEDD	HR = 0.75 (0.25–2.25), p = 0.60	HR = 1.91 (0.94–3.89), p = 0.07
CO	HR = 1.19 (0.37–3.78), p = 0.77	HR = 0.98 (0.66–1.46), p = 0.92
